# Eicosapentaenoic acid in emotional well-being and brain aging: evidence boundaries, clinical heterogeneity, and a peripheral-to-central resolution-biology framework

**DOI:** 10.3389/fnut.2026.1878417

**Published:** 2026-08-07

**Authors:** Qihui Peng

**Affiliations:** Foresight Life Science Pte. Ltd., Singapore, Singapore

**Keywords:** blood-brain barrier, brain aging, depression, eicosapentaenoic acid, emotional well-being, EPA, E-series resolvins, inflammaging

## Abstract

Emotional well-being and brain aging are increasingly understood as overlapping outcomes shaped by inflammatory, neuroimmune, vascular, and metabolic processes. This structured critical narrative review evaluates eicosapentaenoic acid (EPA) as a mechanistically distinct omega-3 fatty acid while explicitly separating EPA-specific evidence from mixed omega-3, fish-intake, and biomarker-status evidence. Particular attention is given to EPA-derived specialized pro-resolving mediators, especially E-series resolvins, and to the hypothesis that EPA may influence brain-relevant outcomes through peripheral inflammatory resolution, endothelial signaling, blood-brain barrier function, neurovascular pathways, and the peripheral immune-brain axis. The current evidence is heterogeneous and differs substantially in directness. EPA-predominant evidence is most developed for depressive symptoms, whereas brain-aging and imaging evidence is often based on fish intake, total omega-3 exposure, or red blood cell EPA+DHA status rather than isolated EPA intervention. This review therefore interprets such evidence as hypothesis-generating rather than as proof of EPA-specific effects. Differences in EPA dose, EPA:DHA ratio, formulation, placebo choice, duration, background diet, baseline omega-3 status, baseline inflammation, clinical phenotype, concomitant treatment, and endpoint selection are examined as potential sources of heterogeneity. The review proposes a testable peripheral-to-central resolution-biology framework for future biomarker-informed research, not a validated clinical decision pathway or universal intervention. EPA is best evaluated as a candidate research exposure whose relevance depends on biological context, verified exposure, inflammatory phenotype, and mechanism-aligned outcomes.

## 1 Introduction

Emotional well-being and brain aging are often discussed as separate domains, yet they overlap at biological and functional levels. Aging-related changes in inflammatory tone, vascular integrity, metabolic regulation, stress responsiveness, and neuroimmune signaling may influence both mood-related outcomes and cognitive trajectories. In older adults, depressive symptoms, fatigue, sleep disruption, reduced motivation, and subtle cognitive decline may appear before clearly defined neuropsychiatric or neurodegenerative disease. This creates a rationale for examining shared biological pathways rather than treating mood and brain aging as isolated endpoints.

Long-chain omega-3 polyunsaturated fatty acids have received sustained attention in this area. However, broad omega-3 terminology can obscure important mechanistic and clinical distinctions. EPA and docosahexaenoic acid (DHA) differ in tissue distribution, lipid mediator biology, and likely roles in brain-related outcomes. DHA is often emphasized as a neural structural fatty acid, whereas EPA may be more relevant to inflammatory resolution, lipid signaling, vascular-endothelial function, and peripheral immune regulation. For this reason, EPA should be evaluated on its own terms while avoiding attribution of mixed omega-3 evidence to EPA alone.

EPA is particularly relevant to a resolution-biology framework. Beyond the broad language of anti-inflammatory effects, EPA can serve as a precursor to specialized pro-resolving mediators, including E-series resolvins. These lipid mediators frame EPA not simply as an inhibitor of inflammation, but as a substrate linked to active resolution processes. This distinction is important for emotional well-being and brain aging because persistent low-grade inflammation, microglial priming, vascular dysfunction, and metabolic-inflammatory burden may contribute to clinical vulnerability in selected populations.

A further challenge concerns how EPA may influence brain-relevant outcomes. EPA does not need to act solely through bulk accumulation in brain tissue. Peripheral inflammatory resolution, endothelial signaling, blood-brain barrier regulation, neurovascular unit function, and peripheral immune-brain communication provide plausible routes through which EPA-related biology could influence brain-related outcomes. However, these pathways should be framed as testable hypotheses, because direct EPA-specific human intervention evidence for several brain-level endpoints remains limited.

Clinical evidence remains heterogeneous. Some meta-analyses and guidelines suggest that EPA-predominant formulations may be more relevant to depressive symptoms than DHA-predominant or low-EPA mixed formulations (1–7). At the same time, large prevention trials and systematic reviews in broadly recruited or clinically complex populations have reported null or mixed outcomes (8–11). These findings should not be treated as contradictory fragments. Instead, they highlight the need to examine biological stratification, baseline omega-3 status, dose, formulation, inflammatory phenotype, placebo choice, concomitant treatments, and endpoint selection.

This review adopts a structured critical narrative approach. Its central argument is that EPA should not be evaluated as a universal intervention for emotional well-being or brain aging. Rather, EPA is evaluated as a mechanistically distinct research exposure that may be most relevant in subgroups characterized by low omega-3 status, low-grade inflammation, metabolic-inflammatory burden, depressive symptoms, or aging-related neuroimmune vulnerability. The review also explicitly distinguishes EPA-specific evidence from mixed EPA/DHA, fish-intake, and omega-3-status evidence, particularly in the brain-aging literature.

This review does not aim to establish EPA as a universal intervention for mood or brain-aging outcomes. Rather, it examines whether EPA may be more biologically plausible in selected inflammatory, neuroimmune, or low omega-3-status phenotypes, and how future studies might test this hypothesis with exposure verification, biomarker-informed stratification, mechanism-aligned endpoints, and transparent reporting of null evidence.

Unlike previous omega-3 reviews that primarily evaluate clinical efficacy, combine EPA and DHA as a single exposure category, or focus on depression and cognition as separate outcomes, this review specifically reframes EPA through a peripheral-to-central resolution-biology model. The central contribution is not to claim broad EPA efficacy, but to use mechanistic pathways, null evidence, clinical heterogeneity, and attribution boundaries to generate testable hypotheses for emotional well-being and brain aging.

### 1.1 Review aim and research questions

The aim of this review is to critically evaluate EPA as a mechanistically distinct omega-3 fatty acid in relation to emotional well-being and brain aging, with particular attention to mechanisms, population heterogeneity, biomarkers, imaging evidence, and translational gaps. The review is guided by five questions:

- What mechanistic pathways plausibly link EPA to emotional well-being and brain aging?
- How do EPA-derived lipid mediators, especially E-series resolvins, contribute to a resolution-biology framework?
- To what extent might EPA act through peripheral inflammatory resolution and the peripheral immune-brain axis rather than direct central accumulation alone?
- Which clinical and biological factors may explain heterogeneous outcomes across EPA, EPA-predominant, and mixed omega-3 studies?
- How can omega-3 status, inflammatory biomarkers, lipid mediator profiling, and brain imaging improve future EPA research?

### 1.2 Central argument

EPA should be understood not merely as a general omega-3 fatty acid, but as a mechanistically distinct lipid mediator precursor whose relevance to emotional well-being and brain aging may depend on inflammatory context, omega-3 status, neuroimmune vulnerability, and design sensitivity in clinical studies. This argument is hypothesis-generating and should not be interpreted as clinical implementation guidance.

## 2 Methods and evidence-synthesis approach

This article was designed as a structured critical narrative review rather than a formal systematic review or meta-analysis. The objective was not to pool all available effect sizes, but to integrate mechanistic, clinical, biomarker, imaging, safety, and translational evidence relevant to EPA, emotional well-being, and brain aging. The structured elements of the approach improve transparency, reproducibility of the search logic, and critical interpretation of heterogeneous evidence. No protocol was registered, and no formal risk-of-bias scoring or GRADE-style certainty rating was undertaken.

### 2.1 Search sources, database-specific strategies, and final search date

Literature searches were conducted in PubMed/MEDLINE, Scopus, Web of Science Core Collection, and Google Scholar, supplemented by backward and forward reference chaining from relevant guidelines, systematic reviews, meta-analyses, and randomized trials. The final structured search update was completed on 30 April 2026. A subsequent citation and reference verification in July 2026 did not expand the evidence base or introduce new claims from literature outside the structured search.

Six prespecified concept groups were addressed: (1) EPA and emotional well-being or depression; (2) EPA or omega-3 exposure and brain aging, cognition, or imaging; (3) EPA and specialized pro-resolving mediators; (4) EPA or omega-3 exposure and inflammatory or neuroimmune markers; (5) EPA or omega-3 exposure and blood-brain barrier, neurovascular, or peripheral-to-central signaling; and (6) safety and tolerability. The retained record contains the generic core strings. Supplementary Table S1 provides database-specific reproducible translations with explicit field restrictions; because contemporaneous per-database query histories were not preserved, these are labeled as reconstructed implementations rather than verbatim historical run logs.

No contemporaneous database exports or per-database query histories were retained. Consequently, database-level filter settings and the number of Google Scholar results screened per query cannot be reconstructed retrospectively and are reported as unavailable rather than estimated.

### 2.2 Inclusion and exclusion criteria

Eligible sources were peer-reviewed randomized controlled trials, meta-analyses, systematic reviews, practice guidelines, cohort studies, mechanistic human studies, biomarker-imaging analyses, and high-quality mechanistic or preclinical reviews relevant to EPA or omega-3 exposure and at least one review domain. Priority was given to sources that reported or permitted extraction of EPA dose, DHA dose, EPA:DHA ratio, intervention form, comparator, concomitant treatment, baseline inflammation, baseline omega-3 status, achieved exposure, lipid mediators, clinical or imaging outcomes, adverse events, or prespecified subgroup analyses. Exclusions were non-peer-reviewed promotional materials; records unrelated to EPA or omega-3 exposure or to the review domains; duplicate reports when a more complete publication was available; and mechanistic studies without a reasonable link to mood, brain aging, inflammation, resolution biology, or neurovascular interpretation. Screening was conducted by one reviewer because this was a structured narrative review rather than a systematic review.

A structured evidence matrix was used to retain the extracted evidence. The final matrix contained 28 categorized evidence entries representing 27 unique citations; the Kang et al. VITAL-Cog publication appeared in both the brain-aging and anti-evidence categories. Of the 28 entries, 24 included a PMID and 20 included a DOI, and every entry had at least one stable bibliographic identifier. These figures describe the curated evidence set and must not be interpreted as total database retrieval or screening counts.

### 2.3 Screening, study selection, and audit limitations

Titles and abstracts were screened for relevance to the review questions, followed by full-text assessment of sources most directly relevant to clinical outcomes, mechanistic plausibility, biomarker interpretation, safety, and evidence boundaries. When multiple meta-analyses overlapped, the most recent, clinically informative, or methodologically transparent reviews were prioritized, while earlier influential meta-analyses were retained when they addressed EPA dose, EPA predominance, or depression-specific response patterns not fully captured by newer summaries. Overlapping reviews were not counted as independent evidence of efficacy; rather, they were used to map consistency, controversy, and heterogeneity across the field.

Mixed EPA/DHA studies were handled by attribution level. EPA-specific or EPA-predominant studies were interpreted as more direct for EPA hypotheses. Mixed EPA/DHA supplementation, marine omega-3 trials, fish-intake studies, and RBC EPA+DHA biomarker studies were retained when relevant to brain aging or omega-3-status interpretation, but they were not treated as proof of EPA-specific effects. This attribution rule was applied throughout the clinical, brain-aging, mechanistic, and biomarker sections.

The retained evidence matrix was designed for evidence extraction rather than as a prospective screening-flow log, and no contemporaneous database exports were retained. Consequently, the available record does not permit reliable reconstruction of per-database hit counts, database-level duplicate removal, title and abstract exclusions, full-text exclusions by reason, or Google Scholar screening depth. No PRISMA-style flow is claimed. Supplementary Table S1 distinguishes recoverable evidence-set counts from non-recoverable retrieval-flow fields and prevents the curated set of 28 entries from being misreported as the number of records identified by the searches.

### 2.4 Operational scope and endpoint domains

For the purpose of this review, emotional well-being is used as an umbrella construct rather than as a single psychometric endpoint. It includes depressive symptoms, mood-related distress, affective regulation, fatigue or resilience-related domains, and quality-of-life outcomes when these are linked to omega-3, EPA, inflammation, or nutritional intervention evidence. Brain aging is likewise used as a multidimensional construct that includes aging-related cognitive performance, neurovascular function, neuroinflammatory tone, structural or functional imaging markers, and resilience to inflammatory or metabolic stress, rather than as a single diagnostic category.

These domains are not treated as directly interchangeable outcomes. Depression scales, quality-of-life measures, global cognitive tests, imaging markers, and inflammatory biomarkers are interpreted within their respective domains and are not pooled conceptually as a single unified endpoint. Operational examples include HDRS/HAM-D, MADRS, BDI, PHQ-9, HADS or STAI, quality-of-life measures, MMSE, MoCA, executive-function and memory tests, hippocampal volume, white matter hyperintensities, cerebral blood flow, PET-based neuroinflammation markers, and related biomarker-imaging readouts.

### 2.5 Evidence directness, weighting, and limits of inference

Evidence was interpreted according to study design, directness, and relevance to the review questions. Meta-analyses, randomized controlled trials, and practice guidelines were given greater weight for clinical outcome interpretation, while mechanistic human studies were used to support pathway engagement. Observational studies, biomarker-imaging analyses, cell or animal models, and mechanistic reviews were used primarily to support biological plausibility and hypothesis generation. Larger randomized studies, EPA-predominant or EPA-specific exposure, verified blood fatty acid response, biomarker stratification, and prespecified clinical endpoints were considered stronger sources of inference than unstratified mixed omega-3 studies or preclinical models.

Mechanistic plausibility is not treated as proof of clinical efficacy. Null and mixed trials are retained as boundary-setting evidence, not as findings to be dismissed. Because the review is narrative and mechanism-oriented, its conclusions remain subject to selection, interpretive, and narrative-synthesis bias. The resulting framework should therefore be read as a conceptual and hypothesis-generating synthesis rather than as a pooled estimate of clinical efficacy, a systematic certainty-of-evidence assessment, or a validated clinical decision pathway. The evidence-directness framework used for interpretation is summarized in Table 1.

**TABLE 1:** Evidence-directness framework used for interpretation.

Evidence level	Definition	Use in this review	Boundary
EPA-specific human intervention evidence	Isolated or clearly EPA-predominant intervention with reported EPA exposure and clinical or biomarker outcomes.	Most direct for EPA hypotheses, especially when blood EPA response is verified.	Still limited by trial size, population selection, endpoint sensitivity, and replication.
Mixed EPA/DHA human evidence	Marine omega-3, fish-oil, or EPA+DHA supplementation trials with variable ratios and forms.	Relevant to omega-3 nutrition and clinical heterogeneity.	Cannot be attributed to EPA alone unless EPA dose, ratio, and exposure response support that interpretation.
Fish intake or omega-3-status evidence	Dietary fish, total omega-3 intake, Omega-3 Index, or RBC EPA+DHA associations with clinical or imaging endpoints.	Useful for brain-aging biomarker hypotheses and exposure-status frameworks.	Associational and usually not EPA-specific; may reflect DHA, diet quality, health behaviors, or confounding.
Mechanistic or preclinical evidence	Cellular, animal, lipid mediator, neuroimmune, BBB, glial, or neurovascular studies.	Supports biological plausibility and identifies measurable pathways for future trials.	Does not establish clinical benefit or EPA-specific human causality.
Conceptual inference	Integration across pathways, clinical heterogeneity, null trials, and mechanistic plausibility.	Generates testable hypotheses and study-design recommendations.	Must remain falsifiable and should not be used to explain all null findings post hoc.

## 3 EPA biology and mechanistic distinction

A central premise of this review is that EPA should be separated analytically from broad omega-3 terminology. Many clinical and epidemiological studies use mixed omega-3 supplements or dietary fish intake as exposure variables, making it difficult to isolate EPA-specific mechanisms. This limitation is particularly important in brain-related outcomes, where DHA and EPA may contribute through different biological roles.

EPA can be incorporated into cell membranes, compete with arachidonic acid-derived inflammatory pathways, alter eicosanoid balance, and serve as a substrate for specialized pro-resolving mediators. These properties make EPA relevant to inflammatory resolution, endothelial function, immune regulation, and lipid mediator signaling. The biological interpretation of EPA studies therefore depends not only on nominal dose but also on chemical form, EPA:DHA ratio, duration, adherence, baseline omega-3 status, and achieved blood EPA levels.

EPA’s relevance to brain aging should be framed cautiously. Because EPA may not accumulate in brain tissue to the same extent as DHA, its brain-relevant effects may involve a proposed sequence from EPA exposure and EPA-derived lipid mediators to peripheral inflammatory-resolution pathways and candidate peripheral-to-central routes. These routes include vascular, humoral, neural, and endothelial communication, with the BBB and neurovascular interface representing a candidate bridge to brain-level responses. This framework is biologically plausible but remains unevenly validated in EPA-specific human trials.

## 4 Mechanistic pathways: from EPA-derived lipid mediators to the peripheral-brain axis

EPA and DHA should not be treated as interchangeable. DHA is more strongly associated with neural membrane structure, whereas EPA may be more relevant to lipid signaling, inflammatory resolution, and peripheral immune regulation. This distinction is essential when interpreting clinical trials, because low-dose mixed fish-oil studies cannot be assumed to test the same biological hypothesis as EPA-predominant interventions. This distinction is not intended to position EPA as a replacement for DHA or for comprehensive omega-3 nutrition. DHA retains irreplaceable structural and developmental roles in neural membranes, while EPA is emphasized here because of its distinct lipid mediator, inflammatory-resolution, and peripheral immune-signaling relevance.

A major mechanistic reason to focus on EPA is its role as a precursor of E-series resolvins and other specialized pro-resolving mediators. Resolution biology emphasizes the active termination of inflammatory responses, clearance of inflammatory debris, and restoration of tissue homeostasis. Human supplementation data show that EPA can increase circulating EPA-derived lipid mediator precursors, including 18-HEPE, together with broader shifts in anti-inflammatory and pro-resolving lipid mediator profiles (12, 13). These findings support pathway engagement but do not prove that EPA-derived mediators cause clinical improvement in mood or brain-aging outcomes.

The human evidence connecting EPA supplementation, E-series lipid mediator biology, inflammation reduction, and depressive symptom change remains preliminary. In particular, the Lamon-Fava studies provide valuable mechanistic evidence in a specific major depressive disorder population with elevated inflammation, but they come from the same research group and require independent replication in other inflammatory phenotypes, broader emotional well-being populations, and aging-related cohorts before wider generalization is justified.

The broader resolution-biology rationale is not limited to these two clinical studies. Foundational work on resolvins and protectins, together with neuroinflammation and glial-cell literature, supports the general biological plausibility of active inflammatory resolution as a conserved pathway relevant to immune and neural homeostasis (14–19). However, the specific clinical claim that EPA-derived SPM changes mediate emotional well-being or brain-aging outcomes in humans remains narrower and should be treated as an open empirical question.

**FIGURE 1:**
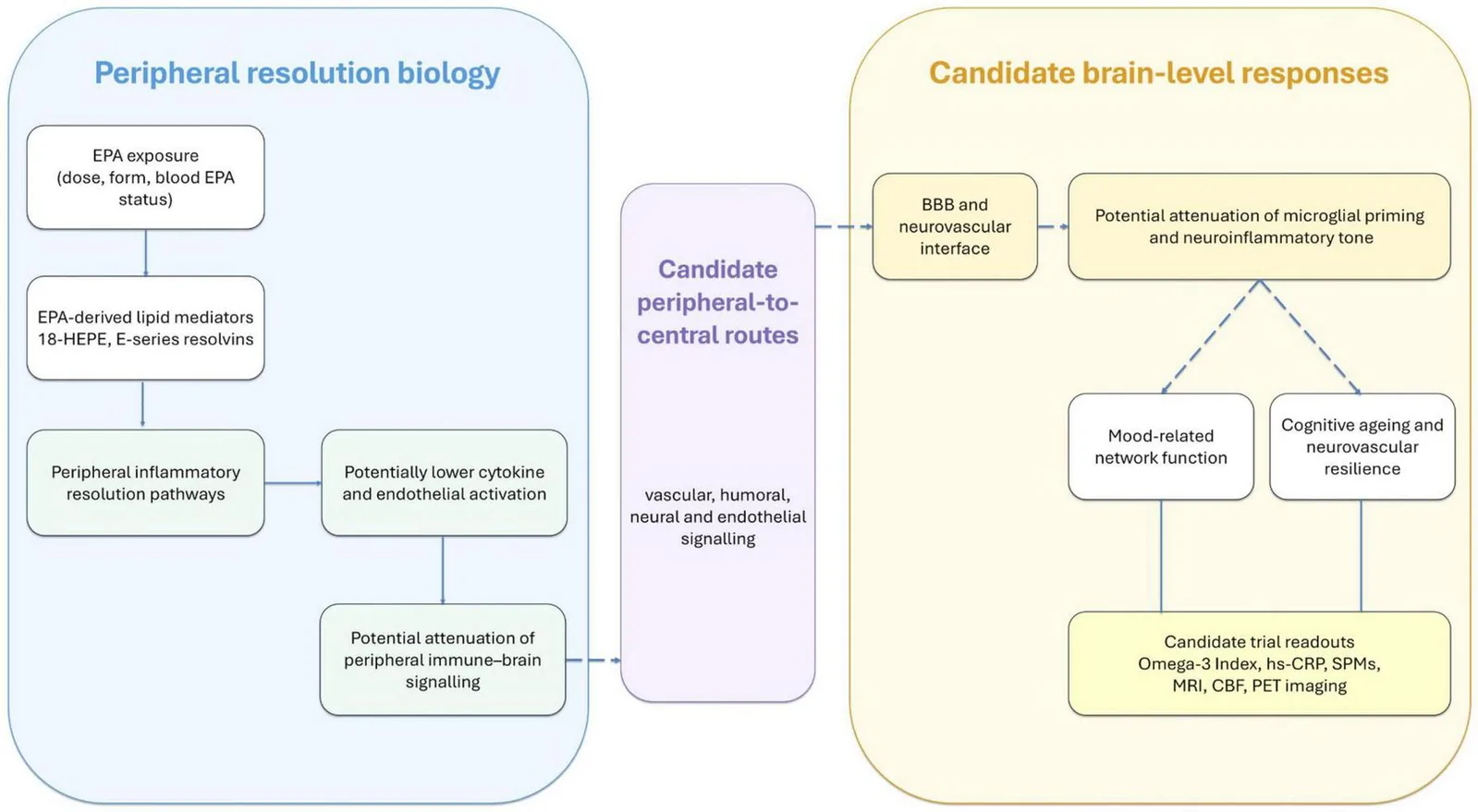
Hypothesis-generating peripheral-to-central resolution-biology framework for EPA. The schematic summarizes proposed links between EPA exposure, EPA-derived lipid mediators, peripheral inflammatory-resolution pathways, candidate peripheral-to-central routes, and candidate brain-level responses relevant to emotional well-being and cognitive aging. Dashed arrows indicate hypothesized, conditional relationships and do not imply established causal effects. Solid arrows within the peripheral panel organize the proposed biological sequence, while the lines to candidate trial readouts denote potential measurement domains; neither signifies that the full pathway has been validated in human intervention studies.

EPA may influence brain-related outcomes through candidate peripheral-to-central routes, but the directness of human evidence differs across pathways. Peripheral inflammation can affect the brain through cytokine signaling, endothelial activation, blood-brain barrier regulation, and neural or humoral immune communication, with possible downstream changes in microglial reactivity. EPA-related inflammatory resolution may therefore modify the biological environment in which mood and cognition are expressed, even when direct central EPA accumulation is limited. Figure 1 presents these links as conditional hypotheses rather than as a demonstrated EPA-specific causal sequence in humans.

The BBB and neurovascular interface provide a candidate bridge between peripheral EPA biology and brain-level responses. At present, however, EPA-specific human evidence directly measuring BBB permeability, cerebral blood flow responses, neurovascular coupling, PET-indexed neuroinflammation, or vagal signaling remains limited. These modalities should be viewed as candidate trial readouts for future validation of the framework rather than as already demonstrated EPA effects.

Microglia link inflammation, mood, cognition, and aging. With aging, microglia may become more reactive to peripheral inflammatory signals, a process often described as microglial priming. The potential attenuation of microglial priming or neuroinflammatory tone by EPA-related pathways remains a hypothesis because direct human evidence is incomplete. The key translational question is whether EPA can measurably shift inflammatory or neuroimmune tone in populations where these pathways are already activated.

Interpretation note: Table 2 summarizes evidence used for mechanistic plausibility, not proof of clinical efficacy. Human lipid mediator evidence remains concentrated in a small number of studies and requires independent replication before broad extrapolation to emotional well-being or brain-aging populations.

**TABLE 2:** Mechanistic evidence linking EPA to inflammatory resolution and peripheral-brain communication.

Evidence source	Evidence level	Main relevance	Main limitation
Lamon-Fava et al., 2021; 2023 (12, 13)	EPA-specific / EPA-predominant human mechanistic evidence	EPA dose-response, 18-HEPE and pro-resolving mediator response, hs-CRP, depressive symptoms in inflammatory MDD.	Small mechanistic studies; related cohorts; not independently replicated across broader brain-aging or emotional well-being populations.
Borsini et al., 2021 (17)	Mechanistic human-cell / clinical-sample context	LOX/CYP450-derived EPA/DHA metabolites; inflammation and human hippocampal neurogenesis relevance.	Preclinical/cellular emphasis; not a definitive EPA clinical trial.
Joffre et al., 2019; Valente et al., 2022 (14, 16)	Resolution-biology / neuroinflammation context	Supports SPM and neuroinflammation framework beyond generic anti-inflammatory language.	Often review/preclinical evidence; human EPA-specific data limited.
Tiberi and Chiurchiu, 2021 (15)	SPMs and glial-cell biology	Supports discussion of glial homeostasis, microglial priming, and repair.	Requires clinical translation into measurable human endpoints.
Pifferi et al., 2021 (21)	BBB / blood-brain interface context	Supports caution that lipid transport and brain-interface mechanisms are complex.	Not EPA intervention evidence.
Serhan and Petasis, 2011 (18)	Foundational resolution biology	Establishes the conceptual distinction between passive anti-inflammatory suppression and active resolution.	Foundational mechanistic evidence rather than EPA-specific neuropsychiatric or brain-aging intervention evidence.

## 5 Clinical evidence in emotional well-being

The clinical evidence linking EPA to emotional well-being is strongest in depression and depressive symptoms, but it remains heterogeneous. Meta-analyses and practice guidelines are most supportive when interventions are EPA-predominant, when EPA dose is adequate, or when participants have clinically relevant depressive symptoms (1–7). This supports a cautious, phenotype-specific hypothesis rather than a general claim that EPA improves all domains of emotional well-being. Complementary Mendelian randomization evidence suggests a possible role for omega-3 fatty acids, particularly EPA, in major depression, although pleiotropic mechanisms and genomic confounding cannot be fully excluded (20). Representative clinical evidence in emotional well-being is summarized in Table 3.

**TABLE 3:** Representative clinical evidence in emotional well-being.

Evidence source	Design / population	Main relevance	Main limitation
Sublette et al., 2011 (1)	Meta-analysis; major depressive episodes	EPA excess associated with stronger signal in depressive episodes.	EPA-predominant signal relevant, but trial populations and formulations varied.
Liao et al., 2019 (2)	Meta-analysis of double-blind RCTs in depression	Supports benefit in selected contexts and EPA-dose subgroup interpretation.	Substantial heterogeneity; inflammatory phenotype generally not used for enrollment.
Guu et al., 2019 (3)	ISNPR practice guideline for MDD	Clinical-context support for pure EPA or EPA-predominant use when omega-3 intervention is considered.	Guideline context; does not replace trial-level evidence or biomarker stratification.
Lu et al., 2024; Norouziasl et al., 2024 (4, 5)	Umbrella review / dose-response meta-analysis	Maps heterogeneity and dose-response patterns across depressive symptom evidence.	Mixed formulations; variable biomarker reporting.
Kong et al., 2025 (7)	Meta-analysis exploring optimized clinical portrait	Supports the idea that response may depend on clinical profile and intervention characteristics.	Responder profiles need prospective validation.
Lamon-Fava et al., 2023 (12)	Inflammatory MDD mechanistic clinical study	Links EPA dose, lipid mediator response, hs-CRP, and depressive symptom change.	Small and mechanistic; requires replication.
Okereke et al., 2021; Appleton et al., 2021 (8, 9)	Large prevention trial / Cochrane review	Important null or mixed evidence limiting universal claims.	Many studies not EPA-specific; broad or heterogeneous populations.
Arsenyadis et al., 2022; Savard et al., 2023 (10, 11)	Cardiometabolic or cancer-related psychological contexts	Shows the difficulty of translating omega-3 interventions across complex disease contexts.	Comorbidity, disease burden, and endpoints may dilute or alter effects.

The first interpretive issue is diagnostic and phenotypic heterogeneity. Major depressive disorder, subclinical depressive symptoms, anxiety-related distress, fatigue, emotional resilience, and quality of life are overlapping but not equivalent outcomes. Trials that combine these endpoints or enroll participants without a clear symptom threshold may dilute effect estimates. Conversely, studies in clinically symptomatic or biologically enriched groups may be more likely to detect benefit if EPA engages the relevant pathway.

The second issue is formulation and attribution. Studies using mixed fish-oil products with substantial DHA content should not automatically be interpreted as EPA studies. EPA dose, DHA co-administration, EPA:DHA ratio, chemical form, and achieved blood EPA status all affect biological exposure. Depression-focused meta-analyses and guidelines repeatedly identify EPA predominance as a potential effect modifier, whereas broad omega-3 trials may obscure EPA-specific signals (1–7).

The third issue is inflammatory context. If EPA’s relevance depends partly on inflammatory resolution, trials that do not measure or stratify baseline inflammation are difficult to interpret. A null result in a low-risk or low-inflammation population does not necessarily refute a role for EPA in inflammatory depressive phenotypes; it may indicate that the enrolled population was not biologically aligned with the proposed mechanism (12, 13, 17, 22).

High-quality null findings remain central to the synthesis. VITAL-DEP found no significant preventive effect of marine omega-3 supplementation on depression endpoints in a broad adult population without clinically relevant depressive symptoms at baseline (8). This result limits overgeneralization and argues against a universal mood-prevention narrative. It also highlights the need for symptom threshold, inflammation, and omega-3 status stratification before interpreting negative findings as evidence against all EPA-related hypotheses.

Overall, the emotional well-being literature supports a targeted rather than universal interpretation. EPA-rich interventions appear most plausible in subgroups with depressive symptoms, low omega-3 status, elevated inflammatory tone, metabolic-inflammatory burden, or other features suggesting biological responsiveness. This interpretation is more defensible than claiming broad efficacy across all mood-related outcomes and is consistent with both supportive and null evidence (1–11).

## 6 EPA and brain aging

Brain aging is a multidimensional process involving cognition, vascular function, neuroimmune activity, metabolic regulation, structural brain integrity, and resilience to stress or inflammatory insults. EPA’s potential relevance to brain aging should therefore not be reduced to whether supplementation improves global cognitive scores. A more useful question is whether EPA-related mechanisms could influence biological processes that contribute to aging-related cognitive and emotional vulnerability.

Compared with the depression literature, the evidence base for EPA and brain aging is less EPA-specific. Many studies evaluate fish intake, total omega-3 intake, combined EPA/DHA supplementation, or red blood cell EPA+DHA status rather than isolated EPA. This creates an attribution problem: associations with brain volume, hippocampal volume, white matter integrity, or cognitive outcomes should be interpreted as omega-3-status evidence rather than proof of EPA-specific efficacy (23–29).

Nevertheless, these studies remain useful when interpreted as biomarker and hypothesis-generating evidence. Observational analyses linking higher omega-3 status to more favorable MRI or cognitive markers suggest that blood fatty acid status may be relevant to brain-aging trajectories (26–28). However, they do not establish that EPA supplementation alone prevents cognitive aging, nor do they identify which populations are most likely to respond.

Mechanistically, EPA may be more relevant to brain aging through inflammatory resolution, endothelial signaling, neurovascular function, and peripheral immune-brain communication than through direct structural incorporation into neural membranes. This pathway-level claim is supported mainly by mechanistic and biomarker evidence, not by definitive EPA-specific brain-aging trials (12–17, 21, 22).

The mood-cognition interface is especially important in older adults. Depressive symptoms, fatigue, sleep disruption, and inflammatory activation may affect cognitive performance and quality of life before overt dementia or major neurodegenerative disease. EPA may therefore be most appropriately studied in early or intermediate aging-related vulnerability states, where inflammatory and vascular mechanisms remain modifiable.

Current evidence is insufficient to conclude that EPA broadly prevents brain aging. It does, however, justify a focused research hypothesis: EPA may influence brain-aging trajectories in biologically defined subgroups where inflammation, vascular dysfunction, low omega-3 status, and neuroimmune vulnerability intersect (14–17, 21–29).

Interpretation note: Table 4 intentionally separates brain-aging plausibility from EPA-specific causality. Much of the imaging and cognition literature uses mixed omega-3 exposure or RBC EPA+DHA status rather than isolated EPA interventions.

**TABLE 4:** Brain aging, cognition, biomarker, and imaging evidence relevant to EPA interpretation.

Evidence source	Design / population	EPA specificity or biomarker	Interpretation boundary
Shahinfar et al., 2025; Barros et al., 2025 (23, 24)	Systematic review / overview evidence	Often total omega-3 rather than isolated EPA.	Useful for mapping cognitive evidence and heterogeneity, but not EPA-specific.
Kang et al., 2022, VITAL cognition (25)	Large RCT ancillary cognition study	Marine omega-3; not EPA-specific.	High-quality null/mixed evidence for broad prevention populations.
Rouch et al., 2022 (26)	ADNI observational biomarker-imaging analysis	Erythrocyte omega-3 fatty acids.	Supports blood fatty acid status as a brain-aging biomarker framework.
Pottala et al., 2014 (27)	WHIMS-MRI observational analysis	RBC EPA+DHA.	Supports Omega-3 Index / RBC status as exposure biomarker, not EPA-specific proof.
Satizabal et al., 2022 (28)	Framingham Heart Study	RBC omega-3 fatty acids.	Supports integration of blood fatty acids with brain imaging endpoints.
Yang et al., 2024 (29)	MCI systematic review / meta-analysis	Mixed n-3 PUFA evidence.	Useful for cautious discussion of cognitive impairment evidence.

## 7 Clinical heterogeneity and trial-level critical appraisal

Clinical heterogeneity is one of the central features of the EPA literature. It should not be treated simply as inconsistency. Instead, heterogeneity may reveal the variables that determine whether EPA can produce measurable biological or clinical effects. The key question is not whether EPA works universally, but under what conditions EPA is most likely to matter.

EPA trials vary in dose, molecular form, DHA co-administration, ratio, intervention matrix, and achieved exposure. These variables can alter absorption, blood fatty acid response, downstream lipid mediator production, and attribution of effects. EPA formulation should be treated as a methodological variable rather than a commercial claim. From a trial-design perspective, the key issue is not whether one form is universally superior, but whether the intervention reliably produces the intended blood EPA response and permits appropriate EPA-specific interpretation.

Baseline inflammation is a plausible effect modifier. EPA may be more biologically relevant in individuals with low-grade inflammation, inflammatory depressive phenotypes, metabolic-inflammatory burden, or aging-related inflammaging. In broadly healthy populations with low inflammatory tone, there may be less biological opportunity for EPA-mediated resolution pathways to generate measurable clinical change (12, 13, 17, 22).

Baseline omega-3 status is another underused explanatory variable. Individuals with low baseline EPA or low Omega-3 Index may be more likely to show a biological response to supplementation than those with adequate status. Without baseline and achieved fatty acid measures, negative results may reflect insufficient exposure contrast rather than true lack of efficacy (26–28).

Background diet and placebo selection can also shape interpretation. Dietary fish intake, omega-6/omega-3 balance, habitual fat quality, and Mediterranean-style versus Western dietary patterns can influence the incremental contrast produced by supplementation. Placebo oils are not necessarily biologically inert; corn, soybean, olive, mineral, or other oils may differ in metabolic and inflammatory relevance. Placebo choice should therefore be reported and considered when interpreting both null and positive trials.

Concomitant antidepressant use, symptom threshold, study size, and endpoint sensitivity further influence trial interpretation. Adjunctive trials and monotherapy trials test different clinical questions. Trials enrolling participants with minimal symptoms, broad prevention populations, or heterogeneous comorbidities may be less likely to detect a pathway-specific effect. Small trials may be vulnerable to imprecision, while meta-analyses may be shaped by between-study heterogeneity, selective reporting, publication bias, and subgroup instability.

Null and mixed findings are essential to a credible critical review. Large prevention trials and systematic reviews show that marine omega-3 supplementation does not consistently prevent depression or cognitive decline in broadly recruited populations (8–11, 25). These findings do not support a universal EPA narrative. They instead point to design questions: Was the population biologically enriched? Was baseline inflammation measured? Was baseline omega-3 status low? Was EPA exposure adequate? Was the placebo appropriate? Were endpoints sensitive to the proposed mechanism? Selected pivotal trials and meta-analyses illustrating these sources of heterogeneity are summarized in Table 5.

**TABLE 5:** Selected pivotal trials and meta-analyses illustrating formulation, phenotype, exposure verification, comparator choice, quantitative effects, and evidence heterogeneity in EPA and marine omega-3 studies.

Study; evidence set, sample/phenotype, and setting	Exposure, comparator, formulation, and duration	Baseline biomarkers, achieved exposure, or appraisal variables	Principal effect estimate	Heterogeneity, bias/certainty, and major limitation
Liao et al., 2019 (2); meta-analysis of 26 double-blind placebo-controlled trials (*n* = 2,160) across depressive disorders and depressive-symptom populations.	EPA-pure, EPA-major (≥60% EPA), DHA-pure, and DHA-major formulations; dose, duration, placebo oils, and monotherapy/adjunctive settings varied.	Baseline inflammation and omega-3 status were not consistently available; achieved fatty-acid exposure was not used as a pooled stratifier.	Overall SMD -0.28 (95% CI -0.47 to -0.09). At EPA ≤ 1 g/day, EPA-pure and EPA-major subgroup SMDs were -0.50 (*p* = 0.003) and -1.03 (*p* = 0.03).	Substantial heterogeneity (I^2^ = 75%); Egger *p* = 0.17; no formal certainty grade. Small subgroups and formulation/population heterogeneity limit EPA-specific inference.
Norouziasl et al., 2024 (5); 67 RCTs; 53 trials (*n* = 10,167) contributed depression-severity data across populations with and without established depression.	Mixed n-3 interventions with variable EPA/DHA composition, dose, duration, placebo, and concomitant-treatment contexts; dose-response analyses were reported.	Baseline inflammation, omega-3 status, and achieved exposure were inconsistently reported; no credible subgroup difference by EPA vs. DHA vs. combined formulation.	Depression risk: OR 0.95 (95% CI 0.79 to 1.15; GRADE moderate). Per 1 g/day, symptom severity: SMD -1.38 (95% CI -1.69 to -1.07; GRADE moderate).	Very high heterogeneity for severity (I^2^ = 97%) and evidence of publication bias (Egger *p* = 0.01; Begg *p* = 0.001); pooled estimates are not EPA-specific.
Appleton et al., 2021 (9); Cochrane review of 35 studies in adults with major depressive disorder; 34 placebo-controlled studies (*n* = 1,924) and one antidepressant comparison (*n* = 40).	n-3 PUFA formulations, EPA/DHA composition, dose, duration, and placebo varied; most evidence compared supplementation with placebo.	Baseline inflammation, omega-3 status, and achieved exposure were not consistently available for stratified interpretation.	Depressive symptoms: SMD -0.40 (95% CI -0.64 to -0.16; 33 studies, *n* = 1,848), equivalent to about 2.5 HDRS points and below the 3-point minimal clinically important difference.	Considerable heterogeneity; funnel/sensitivity analyses suggested possible positive bias; very-low-certainty evidence. The average effect was unlikely to be clinically meaningful.
Wu et al., 2024 (6); *n* = 60 adults with major depressive disorder (30 per arm); pilot monotherapy RCT with no antidepressants or psychotherapy during the trial.	EPA 2.1 g/day plus DHA 1.1 g/day vs. soybean oil 3.2 g/day; 12 weeks.	Not inflammation-enriched. Baseline erythrocyte EPA was 0.67% vs. 0.45%; week-12 EPA was 1.46% vs. 1.75% (active vs. placebo), with no between-group difference (*p* = 0.60).	Hamilton Rating Scale for Depression scores favored omega-3 at weeks 4, 6, 8, and 12; response and remission differences were not significant.	Small pilot; mixed EPA/DHA exposure and weak between-group biomarker contrast limit EPA-specific attribution.
Mischoulon et al., 2019 (30), with the Lamon-Fava et al., 2023 mechanistic analysis (12); *n* = 61 unmedicated adults with major depressive disorder, body mass index >25 kg/m^2^, and hs-CRP ≥ 3 mg/L.	EPA-only 1, 2, or 4 g/day vs. soybean oil; 12 weeks.	Inflammatory phenotype was required; baseline omega-3 status was not an enrollment criterion. Plasma EPA and 18-HEPE increased with dose and time in 42 completers.	Response at 4 g/day was 64% vs. 40% with placebo (OR 2.63; d = 0.53), but was not statistically significant; response rates at 1 and 2 g/day were 38% and 36%.	Dose-finding sample and mechanistic subset were modest; mediator-response analyses were secondary and require replication.
Okereke et al., 2021, VITAL-DEP (8); *n* = 18,353 older adults without clinically relevant depression at baseline; population-prevention setting with usual care permitted.	Marine omega-3 1 g/day (EPA 465 mg plus DHA 375 mg) vs. olive-oil placebo; median 5.3 years.	Neither elevated inflammation nor low omega-3 status was required. Achieved EPA or omega-3 exposure was not reported for the depression cohort in a form suitable for responder stratification.	No mood-score benefit; the incident or recurrent depression or clinically relevant symptom endpoint was slightly more frequent with omega-3 (HR 1.13).	Broad prevention phenotype, mixed modest dose, and lack of enrichment do not test an EPA-specific treatment hypothesis.
Kang et al., 2022, VITAL-Cog/CTSC-Cog (25); 3,424 telephone and 794 in-person participants (4,218 total); community adults aged ≥60 years; population-prevention setting with usual care continued.	Marine omega-3 1 g/day (EPA 465 mg plus DHA 375 mg) vs. olive-oil placebo; 2–3 years.	Not enriched for inflammation, cognitive impairment, or low omega-3 status. Achieved exposure was not incorporated into the primary cognitive analyses.	No cognitive benefit; pooled annual change difference -0.01 SD (95% CI -0.02 to 0.003; *p* = 0.15).	Broad healthy population, mixed modest dose, and no mechanism-aligned imaging endpoint limit inference about EPA-specific brain-aging effects.
Savard et al., 2023 (11); *n* = 130 men with localized prostate cancer after radical prostatectomy; secondary psychological-outcome analysis in a cancer-treatment and postoperative-recovery setting.	Monoacylglyceride EPA 3 g/day vs. high-oleic sunflower oil 3.75 g/day; approximately 1 year, beginning 4–10 weeks before surgery.	Neither inflammation nor low omega-3 status was an enrollment criterion. After approximately 7 weeks, RBC EPA was 2.77% vs. 0.79% and prostate-tissue EPA was 1.03% vs. 0.26% (active vs. placebo).	EPA did not produce greater improvement in depression, anxiety, fatigue, insomnia, or cognitive complaints; symptoms improved over time in both groups.	Psychological measures were secondary endpoints, and the disease-specific recovery context constrains generalization.

Interpretation note: Nominal dose alone is insufficient. Population phenotype, EPA/DHA composition, placebo oil, concomitant care, baseline inflammatory and fatty-acid status, achieved exposure, endpoint purpose, heterogeneity, publication bias, and certainty jointly determine what an individual trial or meta-analysis can establish. Meta-analytic estimates are reported with heterogeneity and bias/certainty assessments where available. Supplementary Table S2 provides the corresponding interpretation framework.

## 8 Biomarkers, imaging, and integrated trial design

Biomarkers and brain imaging are essential for connecting EPA mechanisms with heterogeneous clinical outcomes. Symptom scales and global cognitive endpoints are clinically meaningful, but they are downstream measures influenced by many non-biological factors. Biomarkers can help determine whether EPA exposure was achieved, whether the proposed pathway was engaged, and whether clinical non-response reflects inadequate exposure, inappropriate population selection, or true lack of effect.

The Omega-3 Index and related blood fatty acid measures are among the most useful exposure biomarkers in EPA research. Baseline status can identify participants with greater biological room to respond, while post-intervention status can verify adherence and achieved exposure. In brain aging research, associations between red blood cell EPA+DHA status and MRI or cognitive markers support the relevance of fatty acid status as a biomarker, but they do not prove EPA-specific efficacy (26–28).

Inflammatory biomarkers are important because EPA’s strongest biological rationale may apply to populations with low-grade inflammation, inflammatory depression, metabolic-inflammatory burden, or inflammaging. Candidate markers include high-sensitivity C-reactive protein, interleukin-6, tumor necrosis factor-alpha, and broader cytokine or immune-metabolic panels. Baseline inflammatory status should be considered an effect modifier, not merely a descriptive variable.

EPA-derived lipid mediators, including E-series resolvins, are promising but underused biomarkers. They can help move the field beyond broad anti-inflammatory language toward a resolution-biology framework. Human studies that connect EPA supplementation, pro-resolving lipid mediators, and clinical response are valuable, although methods remain technically demanding and are not yet routinely incorporated into neuropsychiatric or aging trials (12, 13).

Brain imaging may bridge peripheral biomarkers and brain-level outcomes. Relevant modalities include structural MRI, diffusion imaging, functional MRI, arterial-spin-labeling MRI for cerebral blood flow, PET-based neuroinflammation markers, neurovascular reactivity, and brain-age algorithms. The current evidence should be interpreted cautiously because much of it involves fish intake, mixed omega-3 exposure, or combined EPA+DHA status rather than EPA-specific interventions (26–29).

Future EPA trials should integrate exposure, mechanism, and brain-level endpoints rather than treating biomarkers as optional add-ons. A biologically informative design would verify EPA exposure, characterize baseline inflammation and omega-3 status, measure lipid mediator changes where feasible, and select candidate trial readouts aligned with the hypothesized pathway. These may include the Omega-3 Index, hs-CRP, specialized pro-resolving mediators, MRI, cerebral blood flow, or PET imaging (12, 13, 21, 26–29).

## 9 Translational gaps, boundary conditions, and future directions

The EPA literature supports biological plausibility but not a universal clinical claim. The translational challenge is to design studies that can determine who is most likely to benefit, through which mechanisms, and under what exposure conditions. Several gaps are particularly important: EPA-specific evidence remains diluted by broad omega-3 frameworks; formulation, dose, and achieved biological exposure are underreported; baseline inflammation and neuroimmune vulnerability are rarely used for stratification; SPMs and E-series resolvins remain mechanistically central but translationally underused; brain-level outcomes remain insufficiently integrated; and clinical endpoints may be too broad or too late.

A mechanism-guided precision-nutrition approach would define the target phenotype first, verify exposure, measure pathway engagement, and select endpoints that match the hypothesized mechanism. Such trials may be smaller but biologically richer, or larger trials with pre-specified stratification by inflammation and omega-3 status. This framework should be used to guide research design and cautious evidence interpretation, not clinical implementation.

To avoid making the precision-nutrition framework unfalsifiable, future EPA studies should prespecify the biological context in which benefit is expected and define what findings would weaken the model. Baseline inflammation, omega-3 status, exposure verification, dose, duration, and outcome selection should be specified before randomization rather than used post hoc to explain null results. The framework would be weakened if adequately powered EPA-predominant trials with verified EPA exposure, sufficient duration, prespecified inflammatory enrichment or stratification, and mechanism-aligned endpoints consistently fail to improve relevant inflammatory, lipid mediator, neurovascular, mood, or cognitive outcomes compared with placebo.

For example, a stringent falsification test would be an adequately powered, preregistered EPA-predominant randomized trial that enrolls participants with prespecified low omega-3 status and inflammatory enrichment, such as an Omega-3 Index below 4% and hs-CRP above 3 mg/L, verifies a meaningful post-intervention exposure response such as an Omega-3 Index approaching or exceeding 8%, uses sufficient duration and adherence monitoring, and still demonstrates no change in peripheral SPMs, inflammatory markers, neurovascular or brain-relevant mechanistic endpoints, or prespecified emotional well-being outcomes compared with placebo (31, 32). Repeated findings of this type would substantially weaken the proposed framework rather than merely indicate that the wrong population had been studied.

A practical extension of the framework is to define enrichment criteria before randomization. This schema is not intended as clinical guidance; rather, it is a trial-design tool for selecting biologically plausible EPA-responsive phenotypes. Candidate enrichment variables include low omega-3 status and elevated low-grade inflammatory tone, while excluding acute inflammatory states or clinical instability. These thresholds should be treated as pragmatic research cut-points to be validated prospectively, not as definitive clinical decision rules.

The proposed framework remains conditional and falsifiable. It would be weakened if adequately powered, preregistered EPA-predominant trials with verified exposure, sufficient duration, prespecified inflammatory and omega-3-status strata, and mechanism-aligned endpoints repeatedly failed to alter resolution-related lipid mediators, inflammatory or neurovascular measures, or prespecified domain-specific outcomes. Supplementary Table S3 presents the full evidence-boundary and validation matrix, including the principal limitations, validation requirements, and findings that would weaken the framework.

## 10 Safety, tolerability, and conflict-of-interest safeguards

Safety and implementation should be considered part of EPA trial design rather than an afterthought. Across nutritional and cardiometabolic research contexts, omega-3 interventions are generally well tolerated, but adverse events and cautions should be reported systematically. Common tolerability issues include gastrointestinal discomfort, nausea, fishy aftertaste or reflux, and adherence challenges.

In high-dose cardiovascular outcome trials, purified EPA and mixed EPA/DHA formulations have raised clinically relevant safety questions, particularly atrial fibrillation/flutter and bleeding-related events (33, 34). These signals are not direct evidence of harm in all nutritional or neuropsychiatric contexts, but they require transparent discussion because they challenge any overly promotional view of high-dose EPA. Populations requiring more careful assessment include individuals using anticoagulant or antiplatelet medications, those with bleeding disorders or planned surgery, individuals with a history of atrial fibrillation or clinically significant arrhythmia, and patients with complex multimorbidity or polypharmacy. These cautions do not negate EPA research but argue for dose justification, product-quality control, adverse-event reporting, and appropriate medical supervision when high-dose or long-duration interventions are tested (3, 5, 33, 34).

The conflict-of-interest context also requires safeguards. Formulation, dose, molecular form, and exposure verification are discussed in this review solely as methodological and pharmacological variables relevant to trial design and evidence attribution. They should not be interpreted as claims for any commercial product, proprietary formulation, or real-world brand implementation. To reduce interpretive bias, the manuscript includes null and mixed evidence, distinguishes EPA-specific evidence from mixed EPA/DHA and fish-intake evidence, separates mechanistic plausibility from clinical efficacy, and frames EPA as a candidate research exposure requiring independent validation.

Follow-up duration also affects interpretation. Short trials may verify blood EPA response or lipid mediator changes but may be insufficient for stable mood, cognitive, neurovascular, or brain-aging outcomes. Conversely, long trials in broadly recruited low-risk populations may dilute effects if baseline symptoms, omega-3 status, and inflammation are not measured. Future studies should therefore prespecify follow-up periods according to the expected biological sequence: exposure verification, pathway engagement, symptom or functional change, and longer-term durability.

## 11 Conclusion

The current literature suggests that EPA should not be interpreted simply as a generic omega-3 nutrient or as an interchangeable component of fish oil. EPA occupies a distinct biological position as a lipid mediator precursor with relevance to inflammatory resolution, peripheral immune regulation, vascular signaling, and potentially brain-relevant outcomes. However, these mechanisms remain unevenly validated in humans and should be interpreted according to the directness and strength of the supporting evidence.

A central message of this review is that heterogeneity should not be reduced to a binary question of efficacy versus inefficacy. Positive, mixed, and null findings may reflect differences in population biology, baseline inflammation, omega-3 status, EPA formulation, achieved exposure, placebo choice, concomitant treatments, and endpoint sensitivity. This is particularly important for emotional well-being and brain aging, where clinical outcomes are shaped by complex interactions among inflammation, vascular function, neuroimmune signaling, mood, cognition, and aging-related vulnerability.

EPA-derived specialized pro-resolving mediators, particularly E-series resolvins, provide a more precise framework than conventional anti-inflammatory language. EPA may influence brain-relevant outcomes not only through direct CNS-related mechanisms, but also through peripheral inflammatory resolution, blood-brain barrier and neurovascular pathways, and peripheral immune-brain communication. Classic work by Serhan and colleagues established the resolution-biology framework for resolvins and protectins, supporting the distinction between passive anti-inflammatory suppression and active resolution of inflammation (18).

Future EPA research should therefore be hypothesis-driven, biomarker-informed, and designed to capture biological responsiveness. Trials should report EPA and DHA doses separately, verify achieved omega-3 status, measure inflammatory and resolution-related biomarkers where feasible, integrate imaging or neurovascular outcomes when brain aging is targeted, and include null evidence in interpretation.

This framework is best understood as a testable, hypothesis-generating model rather than a validated clinical decision pathway. Baseline omega-3 status, inflammatory phenotype, background diet, dose/formulation, and verified biological exposure should guide future research design and cautious interpretation, not routine clinical implementation. Its main contribution is to define where EPA-specific research should become more rigorous: independent replication of lipid mediator findings, direct measurement of BBB and neurovascular endpoints, prespecified inflammatory and omega-3 status stratification, standardized outcome mapping, transparent inclusion of null evidence, and systematic safety monitoring.
