# Supplementary material for: Eicosapentaenoic acid in emotional well-being and brain aging: evidence boundaries, clinical heterogeneity, and a peripheral-to-central resolution-biology framework
Source: Front Nutr. 2026 Aug 7;13:1878417. doi: 10.3389/fnut.2026.1878417 (PMC13495781; doi:10.3389/fnut.2026.1878417)
Supplement: Supplementary file 1 [file Table_1.pdf]

Supplementary Material

Database strategies, retained-record audit, trial-interpretation variables, and evidence boundaries

EVIDENCE-AUDIT NOTE
The retained evidence matrix comprises 28 categorized entries representing 27 unique citations; these are included-evidence counts rather than database retrieval counts.

Supplementary Table S1. Search strategy and retained-record audit

S1A. Database-specific translations of the six core concepts

The retained record contains generic core strings rather than verbatim per-database run histories, and no contemporaneous database exports were retained. The database-specific strings below are reproducible translations reconstructed from those core concepts, with explicit field restrictions. They should not be represented as contemporaneous search exports. Final structured update reported in the manuscript: 30 April 2026. No date restriction is embedded in the strings. Database-level filters and Google Scholar screening depth cannot be reconstructed and are reported as unavailable rather than estimated.

Database	Concept	Reproducible syntax (reconstructed)	Field restriction	Final update	Filters / depth
PubMed/MEDLINE	C1 Mood / emotional well-being	(("eicosapentaenoic acid"[Title/Abstract] OR EPA[Title/Abstract] OR "icosapent ethyl"[Title/Abstract]) AND (depression[Title/Abstract] OR "depressive symptoms"[Title/Abstract] OR mood[Title/Abstract] OR "emotional well-being"[Title/Abstract] OR anxiety[Title/Abstract] OR "quality of life"[Title/Abstract]))	Title/Abstract	30 Apr 2026	Database-level filters were not recorded in the retained matrix.
Scopus	C1 Mood / emotional well-being	TITLE-ABS-KEY(("eicosapentaenoic acid" OR EPA OR "icosapent ethyl") AND (depression OR "depressive symptoms" OR mood OR "emotional well-being" OR anxiety OR "quality of life"))	TITLE-ABS-KEY	30 Apr 2026	Database-level filters were not recorded in the retained matrix.
Web of Science Core Collection	C1 Mood / emotional well-being	TS=(("eicosapentaenoic acid" OR EPA OR "icosapent ethyl") AND (depression OR "depressive symptoms" OR mood OR "emotional well-being" OR anxiety OR "quality of life"))	Topic (TS)	30 Apr 2026	Database-level filters were not recorded in the retained matrix.
Google Scholar	C1 Mood / emotional well-being	("eicosapentaenoic acid" OR EPA OR "icosapent ethyl") AND (depression OR "depressive symptoms" OR mood OR "emotional well-being" OR anxiety OR "quality of life")	Full record; relevance-ranked results	30 Apr 2026	Screening depth was not prospectively logged and cannot be reconstructed from the retained matrix.
PubMed/MEDLINE	C2 Brain aging / cognition / imaging	((EPA[Title/Abstract] OR "eicosapentaenoic acid"[Title/Abstract] OR omega-3[Title/Abstract] OR "n-3 fatty acids"[Title/Abstract]) AND ("brain ageing"[Title/Abstract] OR "brain aging"[Title/Abstract] OR cognition[Title/Abstract] OR "cognitive decline"[Title/Abstract] OR "mild cognitive impairment"[Title/Abstract] OR neuroimaging[Title/Abstract] OR MRI[Title/Abstract] OR "brain volume"[Title/Abstract] OR hippocampus[Title/Abstract]))	Title/Abstract	30 Apr 2026	Database-level filters were not recorded in the retained matrix.
Scopus	C2 Brain aging / cognition / imaging	TITLE-ABS-KEY((EPA OR "eicosapentaenoic acid" OR omega-3 OR "n-3 fatty acids") AND ("brain ageing" OR "brain aging" OR cognition OR "cognitive decline" OR "mild cognitive impairment" OR neuroimaging OR MRI OR "brain volume" OR hippocampus))	TITLE-ABS-KEY	30 Apr 2026	Database-level filters were not recorded in the retained matrix.
Web of Science Core Collection	C2 Brain aging / cognition / imaging	TS=((EPA OR "eicosapentaenoic acid" OR omega-3 OR "n-3 fatty acids") AND ("brain ageing" OR "brain aging" OR cognition OR "cognitive decline" OR "mild cognitive impairment" OR neuroimaging OR MRI OR "brain volume" OR hippocampus))	Topic (TS)	30 Apr 2026	Database-level filters were not recorded in the retained matrix.

Database	Concept	Reproducible syntax (reconstructed)	Field restriction	Final update	Filters / depth
Google Scholar	C2 Brain aging / cognition / imaging	(EPA OR "eicosapentaenoic acid" OR omega-3 OR "n-3 fatty acids") AND ("brain ageing" OR "brain aging" OR cognition OR "cognitive decline" OR "mild cognitive impairment" OR neuroimaging OR MRI OR "brain volume" OR hippocampus)	Full record; relevance-ranked results	30 Apr 2026	Screening depth was not prospectively logged and cannot be reconstructed from the retained matrix.
PubMed/MEDLINE	C3 SPM / resolvin biology	((EPA[Title/Abstract] OR "eicosapentaenoic acid"[Title/Abstract]) AND ("specialized pro-resolving mediators"[Title/Abstract] OR SPM[Title/Abstract] OR resolvin[Title/Abstract] OR "E-series resolvins"[Title/Abstract] OR 18-HEPE[Title/Abstract]))	Title/Abstract	30 Apr 2026	Database-level filters were not recorded in the retained matrix.
Scopus	C3 SPM / resolvin biology	TITLE-ABS-KEY((EPA OR "eicosapentaenoic acid") AND ("specialized pro-resolving mediators" OR SPM OR resolvin OR "E-series resolvins" OR 18-HEPE))	TITLE-ABS-KEY	30 Apr 2026	Database-level filters were not recorded in the retained matrix.
Web of Science Core Collection	C3 SPM / resolvin biology	TS=((EPA OR "eicosapentaenoic acid") AND ("specialized pro-resolving mediators" OR SPM OR resolvin OR "E-series resolvins" OR 18-HEPE))	Topic (TS)	30 Apr 2026	Database-level filters were not recorded in the retained matrix.
Google Scholar	C3 SPM / resolvin biology	(EPA OR "eicosapentaenoic acid") AND ("specialized pro-resolving mediators" OR SPM OR resolvin OR "E-series resolvins" OR 18-HEPE)	Full record; relevance-ranked results	30 Apr 2026	Screening depth was not prospectively logged and cannot be reconstructed from the retained matrix.
PubMed/MEDLINE	C4 Inflammation / neuroimmune	((EPA[Title/Abstract] OR omega-3[Title/Abstract]) AND (inflammation[Title/Abstract] OR hs-CRP[Title/Abstract] OR IL-6[Title/Abstract] OR TNF-alpha[Title/Abstract] OR inflammaging[Title/Abstract] OR neuroinflammation[Title/Abstract] OR microglia[Title/Abstract]))	Title/Abstract	30 Apr 2026	Database-level filters were not recorded in the retained matrix.
Scopus	C4 Inflammation / neuroimmune	TITLE-ABS-KEY((EPA OR omega-3) AND (inflammation OR hs-CRP OR IL-6 OR TNF-alpha OR inflammaging OR neuroinflammation OR microglia))	TITLE-ABS-KEY	30 Apr 2026	Database-level filters were not recorded in the retained matrix.
Web of Science Core Collection	C4 Inflammation / neuroimmune	TS=((EPA OR omega-3) AND (inflammation OR hs-CRP OR IL-6 OR TNF-alpha OR inflammaging OR neuroinflammation OR microglia))	Topic (TS)	30 Apr 2026	Database-level filters were not recorded in the retained matrix.
Google Scholar	C4 Inflammation / neuroimmune	(EPA OR omega-3) AND (inflammation OR hs-CRP OR IL-6 OR TNF-alpha OR inflammaging OR neuroinflammation OR microglia)	Full record; relevance-ranked results	30 Apr 2026	Screening depth was not prospectively logged and cannot be reconstructed from the retained matrix.
PubMed/MEDLINE	C5 BBB / neurovascular / immune–brain	((EPA[Title/Abstract] OR omega-3[Title/Abstract]) AND ("blood-brain barrier"[Title/Abstract] OR BBB[Title/Abstract] OR neurovascular[Title/Abstract] OR endothelial[Title/Abstract] OR "cerebral blood flow"[Title/Abstract] OR "immune-brain axis"[Title/Abstract]))	Title/Abstract	30 Apr 2026	Database-level filters were not recorded in the retained matrix.
Scopus	C5 BBB / neurovascular / immune–brain	TITLE-ABS-KEY((EPA OR omega-3) AND ("blood-brain barrier" OR BBB OR neurovascular OR endothelial OR "cerebral blood flow" OR "immune-brain axis"))	TITLE-ABS-KEY	30 Apr 2026	Database-level filters were not recorded in the retained matrix.
Web of Science Core Collection	C5 BBB / neurovascular / immune–brain	TS=((EPA OR omega-3) AND ("blood-brain barrier" OR BBB OR neurovascular OR endothelial OR "cerebral blood flow" OR "immune-brain axis"))	Topic (TS)	30 Apr 2026	Database-level filters were not recorded in the retained matrix.
Google Scholar	C5 BBB / neurovascular / immune–brain	(EPA OR omega-3) AND ("blood-brain barrier" OR BBB OR neurovascular OR endothelial OR "cerebral blood flow" OR "immune-brain axis")	Full record; relevance-ranked results	30 Apr 2026	Screening depth was not prospectively logged and cannot be reconstructed from the retained matrix.
PubMed/MEDLINE	C6 Safety / tolerability	((EPA[Title/Abstract] OR omega-3[Title/Abstract]) AND (safety[Title/Abstract] OR tolerability[Title/Abstract] OR bleeding[Title/Abstract] OR "atrial fibrillation"[Title/Abstract] OR anticoagulant[Title/Abstract] OR antiplatelet[Title/Abstract]))	Title/Abstract	30 Apr 2026	Database-level filters were not recorded in the retained matrix.

Database	Concept	Reproducible syntax (reconstructed)	Field restriction	Final update	Filters / depth
Scopus	C6 Safety / tolerability	TITLE-ABS-KEY((EPA OR omega-3) AND (safety OR tolerability OR bleeding OR "atrial fibrillation" OR anticoagulant OR antiplatelet))	TITLE-ABS-KEY	30 Apr 2026	Database-level filters were not recorded in the retained matrix.
Web of Science Core Collection	C6 Safety / tolerability	TS=((EPA OR omega-3) AND (safety OR tolerability OR bleeding OR "atrial fibrillation" OR anticoagulant OR antiplatelet))	Topic (TS)	30 Apr 2026	Database-level filters were not recorded in the retained matrix.
Google Scholar	C6 Safety / tolerability	(EPA OR omega-3) AND (safety OR tolerability OR bleeding OR "atrial fibrillation" OR anticoagulant OR antiplatelet)	Full record; relevance-ranked results	30 Apr 2026	Screening depth was not prospectively logged and cannot be reconstructed from the retained matrix.

S1B. What can and cannot be recovered from the retained record

Audit item	Value	Status	Interpretation
Curated evidence entries	28	Recoverable	8 emotional-well-being; 7 brain-aging/imaging; 8 mechanisms; 5 anti-evidence.
Unique citations represented	27	Recoverable	Kang et al. 2022 (PMID 35415212) appears in two evidence categories.
Entries with PMID	24/28	Recoverable	Four entries lack a PMID in the matrix.
Entries with DOI	20/28	Recoverable	Eight entries lack a DOI in the matrix; all entries have at least one stable identifier.
PubMed/MEDLINE records identified	Not recoverable	Not available in retained record	Do not substitute the curated evidence-set count.
Scopus records identified	Not recoverable	Not available in retained record	Do not infer from citation count.
Web of Science records identified	Not recoverable	Not available in retained record	Do not infer from citation count.
Google Scholar records screened	Not recoverable	Not available in retained record	Record the depth per query if the search is rerun.
Additional records from reference chaining	Not recoverable	Not available in retained record	The matrix does not label provenance consistently.
Duplicates removed	Not recoverable	Not available in retained record	The single cross-category duplicate is not a database deduplication count.
Title/abstract exclusions	Not recoverable	Not available in retained record	No prospective screening log was retained with the evidence matrix.
Full texts excluded by reason	Not recoverable	Not available in retained record	Eligibility reasons are defined, but record-level exclusion counts are unavailable.

Supplementary Table S2. Trial-level variables for interpreting EPA and omega-3 clinical evidence

Variable	Why it matters	Recommended interpretation or reporting
EPA dose and achieved exposure	Nominal dose may not produce meaningful blood EPA change if adherence, absorption, or background diet differ.	Report dose and measure baseline/post-intervention Omega-3 Index, RBC EPA/DHA, plasma EPA, or comparable measures.
EPA:DHA ratio and formulation	Mixed EPA/DHA trials may not test an EPA-specific hypothesis; molecular form and matrix affect attribution.	Report EPA and DHA separately; interpret mixed formulations as mixed omega-3 evidence unless EPA predominance and exposure response are clear.
Duration and follow-up	Short trials may show biomarker change without stable clinical effect; long broad trials may dilute effects.	Prespecify timing for exposure verification, pathway engagement, symptom change, and durability.
Placebo choice	Placebo oils may not be metabolically or inflammatory neutral.	Report placebo composition and consider its biological effects when interpreting null or positive outcomes.
Baseline omega-3 status	Adequate baseline status reduces exposure contrast and may dilute effects.	Measure baseline status and prespecify low-status enrichment or subgroup analysis.
Baseline inflammation	Inflammatory phenotype may modify response; diagnostic labels alone may mix mechanisms.	Measure hs-CRP, IL-6, TNF-alpha, or immune-metabolic panels; use prespecified stratification where appropriate.
Concomitant treatment	Adjunctive and monotherapy settings test different hypotheses.	Report antidepressant use, medication stability, comorbidity, and polypharmacy.
Symptom threshold and endpoint sensitivity	Prevention samples, low symptoms, or broad outcomes may miss pathway-specific change.	Define symptom threshold and use domain-specific mood, cognition, quality-of-life, biomarker, and imaging endpoints.
Study size, heterogeneity, and publication bias	Small trials and selective reporting can distort effect estimates; meta-analyses can be sensitive to subgroup definitions.	Interpret effects with sample size, heterogeneity, confidence intervals, and publication-bias assessment in mind.

Note. This table provides the broader interpretive-variable framework used to evaluate exposure, phenotype, comparators, biomarkers, outcomes, and evidence boundaries.

Supplementary Table S3. Evidence boundaries, testability, and falsifiability of the proposed EPA framework

Component	Current evidence status	Boundary / limitation	A priori validation criteria	Findings that would weaken the framework
EPA-derived resolution biology	Human mechanistic evidence shows EPA can increase 18-HEPE and related mediator profiles, especially in inflammatory MDD contexts (12, 13).	Evidence is concentrated in a small number of related studies; broader replication is lacking.	EPA-predominant trials with verified blood response should measure lipid mediators, inflammatory markers, and prespecified outcomes.	Adequately exposed trials repeatedly fail to change resolution-related lipid mediators or inflammatory markers.
Inflammatory phenotype	Inflammation may modify EPA responsiveness, but thresholds are not standardized.	No universal biomarker threshold defines an EPA-responsive phenotype.	Prespecify enrichment or stratification using hs-CRP, IL-6, TNF-alpha, immune-metabolic panels, omega-3 status, or SPM-related markers.	Prespecified inflammatory strata show no differential biological or clinical response despite verified exposure.
BBB, neurovascular, and immune–brain pathways	Evidence is plausible and supported mainly by indirect human biomarker, interface-biology, and preclinical literature.	EPA-specific human trials rarely measure BBB permeability, cerebral blood flow, neurovascular coupling, or direct immune–brain communication.	Include DCE-MRI, ASL-MRI, cerebrovascular reactivity, endothelial markers, autonomic indices, and cytokine or SPM panels.	Verified EPA trials show no change in BBB, neurovascular, endothelial, autonomic, or immune-signaling endpoints.
Mood, cognition, and brain-aging outcomes	EPA-predominant evidence is most developed for depression-related outcomes; brain-aging evidence is less EPA-specific.	Depression, emotional well-being, cognition, quality of life, and imaging markers are not interchangeable.	Prespecify domain-specific instruments and imaging or neurovascular endpoints when brain aging is targeted.	Biomarker-verified EPA-predominant trials repeatedly show no benefit on prespecified outcomes or no relationship between pathway engagement and outcomes.
Precision-nutrition interpretation	The framework integrates formulation, baseline status, inflammation, and mechanism alignment as candidate moderators.	If used only to explain null findings retrospectively, the model becomes weakly falsifiable and biased.	Define target phenotype, exposure criteria, duration, primary outcomes, and subgroup analyses before treatment.	Predefined, biologically enriched and adequately exposed trials consistently fail to show pathway engagement or clinical signals.

Interpretation note. This table prevents overextension by separating plausible pathways from demonstrated human effects and by specifying prospective tests that could weaken the model.

Abbreviations: ASL-MRI, arterial spin-labeling magnetic resonance imaging; DCE-MRI, dynamic contrast-enhanced magnetic resonance imaging.
